# Telemedicine in Afghanistan: Implementation, Outcomes, and Future Directions in a Resource-Constrained Setting

**DOI:** 10.7759/cureus.98822

**Published:** 2025-12-09

**Authors:** Samira Ali, Mohammad Yousuf Sultani, Mahjabin Shahid, Humaira Sadat Sultany, Mohammad Zahid Omerzad, Hashmat Ullah Faizi, Mohammad Ibrahim Sultani, Nafisa Hamidi, Shaqaiq Akhtiyari, Roya Lalzai

**Affiliations:** 1 Obstetrics and Gynaecology, Isteqlal Hospital, Kabul, AFG; 2 Dentistry, Stomatology National and Teaching Hospital, Kabul, AFG; 3 Mental Health, Kabul University of Medical Sciences "Abu Ali Ibn Sina", Kabul, AFG; 4 General Medicine, Kabul University of Medical Sciences "Abu Ali Ibn Sina", Kabul, AFG; 5 General Practice, Nangarhar Medical Faculty, Nangarhar University, Nangarhar, AFG; 6 Medicine, Ankara Yıldırım Beyazıt University, Ankara, TUR; 7 Internal Medicine, Bamyan Provincial Hospital, Bamyan, AFG; 8 Kateb Medical Faculty, Kateb University, Kabul, AFG; 9 Curative Medicine, Kabul University of Medical Sciences "Abu Ali Ibn Sina", Kabul, AFG

**Keywords:** afghanistan, digital health, healthcare access, health systems strengthening, low-income countries, telehealth, telemedicine

## Abstract

Afghanistan faces major challenges in healthcare access due to decades of conflict, geographic isolation, and limited infrastructure. Telemedicine presents a promising solution to bridge these gaps by leveraging technology to connect healthcare providers and patients across distances and time zones. This review explores the current landscape of telemedicine in Afghanistan, synthesizing evidence from published studies, program evaluations, and implementation reports across multiple specialties and healthcare settings. Telemedicine initiatives in the country span critical care, mental health, ophthalmology, general medicine, and emergency services, extending their benefits to both urban and rural populations. Key programs include the Central Asia Health Systems Strengthening (CAHSS) Project (6,140 teleconsultations), tele-ICU services (6,014 consultations showing reduced mortality), mental health interventions in remote provinces, and military teleophthalmology programs. Despite notable barriers such as limited Internet connectivity, unreliable electricity, regulatory gaps, cultural resistance, low digital literacy, and workforce shortages, telemedicine has improved access to care, strengthened provider capacity, reduced stigma, and demonstrated high patient satisfaction and cost-effectiveness, particularly in underserved regions. Overall, telemedicine represents a viable and effective strategy for enhancing healthcare delivery in Afghanistan. Continued progress will depend on sustained investment in digital infrastructure, supportive policy frameworks, workforce development, community engagement, and robust monitoring systems. Further research should focus on longitudinal evaluations, national registries, and comprehensive cost-effectiveness analyses to inform future telemedicine expansion and sustainability.

## Introduction and background

Afghanistan is a low-income country that has endured decades of war, political instability, and widespread poverty, resulting in profound disruptions to its health system and social fabric [1,2]. The population is dispersed across remote and mountainous regions, with more than 30,000 villages and significant barriers to accessing healthcare, particularly outside major urban centers [3]. Health facilities and medical equipment remain inadequate, and the shortage of trained healthcare professionals is acute, especially in rural areas [4,5]. The burden of disease in Afghanistan is substantial, with high rates of both acute and chronic illnesses, including infectious diseases, maternal and child health challenges, and mental health disorders [1,2]. Mental health needs are especially pronounced due to the cumulative effects of conflict, poverty, insecurity, and gender disparities, with large numbers of youth and adults suffering from depression, post-traumatic stress disorder, and other conditions [2]. The COVID-19 pandemic has further strained the health system, highlighting the urgent need for innovative solutions to maintain essential services and reduce infection risk [4,6]. Traditional healthcare delivery models are often impractical in Afghanistan's context due to geographic isolation, security concerns, and infrastructural deficits [3,5]. Patients in remote regions are frequently forced to travel long distances, incurring significant financial and social burdens to access care [7]. Women and other vulnerable groups face additional barriers, though mobile health technologies have shown promise in improving access and acceptability [8]. Telemedicine offers a compelling strategy to address these challenges by leveraging information and communication technologies to connect providers and patients across distances, facilitate specialist consultations, and support capacity building for frontline health workers [3,4,9]. Pilot projects and early implementations in Afghanistan have demonstrated improved access to care, reduced stigma, enhanced provider skills, and increased patient satisfaction, particularly in mental health, critical care, and military medicine [2,10,11]. However, significant barriers remain, including technological limitations, regulatory gaps, and sustainability concerns [6,7]. Given Afghanistan's unique healthcare landscape and the demonstrated potential of telemedicine to improve access and quality of care, there is a clear rationale for conducting comprehensive research on telemedicine implementation, outcomes, and future directions in this context. Such research is essential to inform policy, guide resource allocation, and support the development of scalable, sustainable telemedicine solutions tailored to Afghanistan's needs [3,4].

Search methodology

A comprehensive literature search was performed to identify relevant studies, program evaluations, and implementation reports related to telemedicine in Afghanistan. Major electronic databases, including PubMed, Scopus, Web of Science, and Google Scholar, were systematically searched from their inception to September 2025. The search strategy combined Medical Subject Headings (MeSH) and free-text terms, including “telemedicine,” “telehealth,” “digital health,” “Afghanistan,” and “low-income countries.” Studies were included if they: (1) described or evaluated a telemedicine initiative implemented in Afghanistan or targeting Afghan populations; (2) reported clinical, operational, or patient-centered outcomes; and (3) were available in English. Both quantitative and qualitative studies were considered.

## Review

Current telemedicine programs and specialty coverage

Telemedicine in Afghanistan has evolved into a multifaceted system, leveraging international and local initiatives to address the country's severe healthcare access challenges. This section examines major programs and their specialty coverage across different healthcare domains.

Regional Teleconsultation Networks

The Central Asia Health Systems Strengthening (CAHSS) Project, a collaboration between the Aga Khan Foundation of Canada and the Government of Canada, established a regional hub-and-spoke teleconsultation network. This network connected seven tertiary care facilities with 14 secondary care facilities, providing 6,140 teleconsultations and 52 e-learning sessions to 2,020 staff members between April 2013 and September 2014. The specialties covered included general medicine, pediatrics, surgery, and capacity building, with a focus on minimizing the barriers of distance and time and improving access to low-cost, quality healthcare for rural communities. Ethics and patient rights were explicitly respected during implementation, which is particularly relevant in Afghanistan's sociocultural context [3].

Critical Care Telemedicine

Critical care telemedicine has advanced through the tele-ICU service developed by the Aga Khan University. Between June 2020 and December 2023, this service provided 6,014 teleconsultations to 1,907 patients in 109 medical facilities, including those in Afghanistan. The program initially focused on COVID-19 but expanded to include medical, neonatal, pediatric, and surgical critical care training. The mean duration of teleconsultations was 14.5 minutes, and the service was available 24/7, utilizing two-way audio-visual technology, messaging applications, and telephone calls. The program demonstrated that increased frequency of teleconsultations per patient was associated with a significant reduction in mortality (adjusted odds ratio for >3 consults: 0.28, 95% CI: 0.16-0.48), underscoring the clinical impact of sustained telemedicine engagement in resource-constrained settings [11].

Mental Health Telemedicine

Mental health telemedicine initiatives have targeted depression, psychosis, post-traumatic stress disorder, and substance abuse, particularly in the Badakshan Province. An Android-based mobile application, guided by the World Health Organization Mental Health Gap Action Programme (mhGAP) protocols, enabled community healthcare workers to collect patient information, provide referrals, and access blended learning modules. These interventions improved access to care, reduced stigma, and enhanced service quality, especially in rural and remote areas [2,12].

Military Teleophthalmology

Ophthalmology telemedicine has been piloted in military settings using secure mobile-phone applications. In a prospective case series conducted at 16 military treatment facilities, medics placed teleophthalmology consultations via a mobile application, with responses from an expeditionary ophthalmologist. The mean initial response time was less than four minutes, diagnostic concordance was 86%, and teleconsultation prevented aeromedical evacuation in 14% of cases, enabling 54% of patients to return to duty. All consults were secure and compliant with privacy regulations [10].

Mobile Health Teams (MHTs)

MHTs, supported by the WHO and United Nations Children's Fund (UNICEF), have become central to Afghanistan's strategy for reaching underserved populations, especially since the Taliban's return to power. These teams, which account for 17.1% of health facilities, use mobile services and telemedicine components to deliver maternal and child health, infectious disease, and general outpatient care services. A recent quasi-experimental analysis found that mobile services increased recent healthcare access by approximately 11 percentage points in underserved areas, though the effect diminished with increasing numbers of teams, highlighting the importance of strategic deployment and quality assurance [13].

Military Telemedicine Support

Military telemedicine support has a long history in Afghanistan, with synchronous and asynchronous platforms supporting trauma, surgery, emergency medicine, and general care in operational settings in Afghanistan. These platforms are critical when evacuation is delayed, and they bring advanced expertise to austere environments [9]. The COVID-19 pandemic further accelerated telemedicine adoption, with facilities employing remote consultations, self-care promotion, and patient redirection to alternative sites to maintain essential services [4,14].

Barriers to implementation and adoption

Telemedicine implementation in Afghanistan faces significant technological, infrastructural, regulatory, cultural, financial, and workforce-related barriers that must be addressed to ensure sustainable scaling-up and equitable access.

Technological and Infrastructure Barriers

Poor Internet connectivity, unreliable electricity, and limited access to digital devices are pervasive, especially in rural and remote regions. The CAHSS Project highlighted that isolated communities often lack the necessary infrastructure for teleconsultations, forcing patients to travel long distances and incur substantial financial burdens [3]. Inadequate broadband coverage and unstable power are among the most significant obstacles [6]. The lack of interoperability between systems, the absence of standardized platforms, and insufficient technical support further reduce the effectiveness and utilization of telemedicine services [15,16].

Regulatory and Policy Barriers

Afghanistan lacks a comprehensive national telemedicine policy, and there is limited clarity regarding the legal frameworks for telemedicine practice, data privacy, and patient confidentiality. The absence of clear guidelines for implementation, reimbursement, and liability creates uncertainty for providers and deters adoption [17]. Reimbursement policies are poorly defined, and concerns about data confidentiality are major deterrents, particularly in lower-middle-income countries [18].

Cultural and Digital Literacy Barriers

Cultural resistance and low digital literacy among providers and patients are major obstacles. Traditional expectations of face-to-face care, skepticism about remote consultations, and gender norms, especially in conservative regions, limit telemedicine uptake [19]. Low levels of digital literacy and limited awareness of telemedicine's benefits further compound these challenges [20].

Financial Barriers

Financial constraints affect both health facilities and patients, with the costs of infrastructure, devices, and connectivity often exceeding available resources. Out-of-pocket health expenditures are a major source of financial hardship, with 32% of the population incurring catastrophic health expenditure (defined as 10% of total consumption) [21].

Workforce-Related Barriers

Workforce-related barriers include shortages of trained staff, lack of technical support, and resistance to change in practice. Poor digital literacy and inadequate awareness of digital health technologies among healthcare professionals are common, and the lack of continuous training further impedes adoption [22,23]. These barriers are magnified in rural and remote regions, where infrastructural deficits, financial constraints, and workforce shortages are most severe [3].

Outcomes and Impact

Clinical and Operational Outcomes

Telemedicine interventions in Afghanistan have improved access to specialist care, support for clinical decision-making, and operational efficiency. The CAHSS Project provided over 6,000 teleconsultations and facilitated comprehensive, coordinated care, though specific clinical endpoints such as morbidity or mortality were not reported [3]. The tele-ICU service provided by Aga Khan University showed that increased frequency of teleconsultations per patient was associated with a significant reduction in mortality (adjusted odds ratio for >3 consults: 0.28, 95% CI: 0.16-0.48), with an overall mortality rate of 35.1% among critically ill patients [11]. In mental health, telemedicine interventions in Badakshan province improved access to care, decreased stigma, and enhanced service quality, though quantitative clinical outcomes were not detailed [2,12]. For Afghan refugees in the United States, a culturally adapted, patient navigator-led telehealth program resulted in statistically significant reductions in depression and anxiety symptoms, with large effect sizes (Patient Health Questionnaire-9 (PHQ-9) reduction of 4.44 points, d = 1.05; Generalized Anxiety Disorder 7-item (GAD-7) reduction of 3.70 points, d = 0.94), and was equally effective across genders and age groups [24]. Military teleophthalmology achieved high diagnostic concordance (86%), adherence to clinical guidelines in all cases, and prevented aeromedical evacuation in 14% of cases, enabling 54% of patients to return to duty [10]. MHTs increased recent healthcare access by approximately 11 percentage points in underserved areas, though the effect diminished with increasing numbers of teams [13]. Operationally, telemedicine has facilitated the more efficient use of resources, improved provider capacity, and reduced logistical barriers. The CAHSS Project's hub-and-spoke model supported both teleconsultation and eLearning, with over 2,000 staff benefiting from eLearning sessions [3]. The mean duration of tele-ICU consultations was 14.5 minutes, and increased consultation frequency was associated with improved outcomes [11]. In military settings, the mean initial response time for teleophthalmology consults was under four minutes, and all consults were secure and compliant with privacy regulations [10].

Patient-Centered Outcomes and Equity

Patient-centered outcomes include enhanced access to care, reduced stigma, increased satisfaction, and respect for patients’ rights. The CAHSS Project emphasized ethical implementation and respect for patient rights, contributing to positive patient experiences [3]. In mental health, telemedicine interventions were associated with decreased stigma and improved access, particularly for underserved populations [2,24]. User satisfaction with telemedicine platforms has been high, with median satisfaction scores of 5 out of 5 reported for the teleophthalmology mobile app [10]. In humanitarian settings, mobile health services improved healthcare access, though the relationship between service density and access was complex [13]. The experiences and perspectives of Afghan patients and providers are generally positive, with high satisfaction across specialties and regions. Trust in telemedicine is enhanced by patient-centered communication and reduced by health literacy barriers. Technical challenges, poor internet, device access, and low digital literacy are the main barriers, especially in rural areas [25-27]. Provider satisfaction is high when telemedicine is well-supported and integrated into clinical workflows, but resistance to change and technical limitations persist [28]. Health equity for vulnerable groups, women, children, disabled persons, and ethnic minorities has been promoted through telemedicine by reducing geographic and financial barriers, supporting provider capacity, and integrating it with community-based and humanitarian services. For example, over 90% of women in Nangarhar Province expressed willingness to receive health messages via mobile phones, and MHTs increased access by 11 percentage points in underserved areas [8,13]. However, persistent challenges remain, including infrastructural deficits, digital literacy gaps, and the need for culturally sensitive implementation, particularly for children and vulnerable populations in underresourced settings [29-32].

Integration, policy, and legal-ethical frameworks

Health System Integration

The integration of telemedicine into Afghanistan's national health system is evolving, with most initiatives operating as project-based networks rather than components of a unified national strategy. The CAHSS Project established structured referral pathways between primary, secondary, and tertiary care, enabling timely access to specialist input and reducing the need for patient transfers [3]. Mental health projects in Badakshan province used mobile applications to support referrals, data sharing, and follow-up, enhancing continuity of care [2]. Data sharing remains variable, with some programs incorporating systematic data collection and sharing, while others face challenges due to the lack of standardized electronic health records and limited interoperability. National-level coordination and the development of technical standards are needed to ensure integration of telemedicine data into the wider health information system, requiring system-level enablers and governance frameworks [33,34].

Legal, Ethical, and Data Privacy Frameworks

Afghanistan’s legal, ethical, and data privacy frameworks are characterized by significant gaps and a reliance on international standards. There is no comprehensive national telemedicine law, and most programs operate under the auspices of international organizations and foreign partners. Military telemedicine initiatives use National Institute of Standards and Technology (NIST)-certified encryption and comply with US Department of Defense standards, but civilian programs often use commercial apps lacking robust privacy protections [10,35]. The absence of clear national regulation creates uncertainty for providers regarding malpractice liability, informed consent, and the legitimacy of remote consultations, highlighting the need for clear definitions of telehealth that address research, implementation, and equity considerations [36-38].

Recent Policy Developments

Recent policy developments since 2024 have focused on sustaining and expanding MHTs with support from the WHO and UNICEF. These teams are deployed based on geo-localized needs assessments and supported by digital tools for data collection and service delivery [39]. However, there is no evidence of a new, comprehensive national regulatory framework for telemedicine, and sustainability remains contingent on donor funding and international technical assistance [13].

Cost-Effectiveness and Economic Impact

Cost-effectiveness analyses indicate that telemedicine is generally cost-effective in Afghanistan, particularly when designed to minimize technological complexity and address the needs of remote and underserved populations in the country. Direct and indirect cost savings accrue to both health systems and patients, primarily through reductions in travel, improved resource utilization, and enhanced access to care [39-41]. The breakeven point for telehealth implementation can range from immediate (less than one year) to up to nine years, depending on the modality and scale of deployment [42]. Evidence from systematic reviews demonstrates favorable cost-utility ratios for digital health interventions across diverse healthcare contexts [43]. Out-of-pocket health expenditures are a major source of financial hardship, and telemedicine has the potential to mitigate these costs by reducing the need for travel, accommodation, and lost income [21].

Table 1 summarizes the key cost domains and their impacts [39-42].

**Table 1:** List of domains and their impacts along with their details.

Cost Domain	Health System Impact	Patient Impact
Direct Costs	Hardware, software, training, and maintenance can be minimized with low-tech models	Device/internet purchase, user fees; offset by reduced travel costs
Indirect Costs	Staff time, workflow disruption, and technical support	Time savings, reduced lost productivity, improved access
Direct Savings	Reduced referrals, optimized resource use, and tele-education	Major reduction in travel/accommodation costs, lower out-of-pocket expenditures
Indirect Savings	Improved provider capacity, reduced demand for services	Enhanced access, decreased stigma, improved outcomes
Cost-Effectiveness Summary	Generally favorable, especially for remote/rural care and mobile models; breakeven can be immediate to nine years	Societal savings substantial, especially in underserved areas

Recommendations and future directions

Evidence-Based Recommendations

Evidence-based recommendations for the future development and scaling of telemedicine in Afghanistan emphasize a multi-pronged and context-sensitive approach that addresses infrastructural, regulatory, and social dimensions of healthcare delivery. Any successful initiative must begin with comprehensive needs assessments to systematically evaluate healthcare priorities, technological capacity, and cultural context before program implementation [44]. Building on these assessments, investment in digital infrastructure is essential, with priority given to expanding broadband internet connectivity and ensuring reliable electricity access, particularly in rural and underserved areas [45]. These infrastructural elements must be accompanied by enabling policies and regulatory frameworks that establish clear practice standards, reimbursement mechanisms, liability protections, and data governance measures [45]. Equally critical is strengthening workforce capacity through continuous training programs that enhance healthcare professionals’ competence in using digital health tools and telemedicine platforms [22], while integrating telemedicine services across different specialties and levels of care through well-defined referral pathways and secure data-sharing mechanisms [33]. However, technological and professional capacity alone cannot guarantee success; community engagement must be prioritized to address cultural barriers, increase acceptance, and build public trust by involving local communities in planning and implementation processes [46], while robust data security measures, including encryption methods and privacy protections aligned with international standards, safeguard patient information and promote confidence among both providers and patients [30].

Long-term success requires embedding accountability mechanisms and collaborative governance structures throughout all phases of development. Systematic monitoring and evaluation should be integrated within all telemedicine initiatives, allowing continuous data collection and analysis to inform ongoing refinement and scaling efforts [39]. At the national level, coordination mechanisms must align donor activities, prevent duplication, and enhance sustainability through collaborative governance among key stakeholders [29]. A sustainable implementation strategy, such as the “Initiate-Build-Operate-Transfer” model, should be adopted to ensure long-term local ownership, institutional capacity building, and program continuity, drawing lessons from successful global experiences in comparable resource-limited settings [38,47]. Throughout all phases, equitable development should remain a guiding principle, with special attention given to reducing disparities between urban and rural populations while maintaining consistent quality standards across all regions of Afghanistan [13].

Research Gaps and Future Studies

Long-term clinical and health system outcomes include sustained improvements in access to care, capacity building, and patient-centered outcomes, with evidence of reduced mortality in critical care settings and enhanced mental health service delivery in remote regions [11,48]. However, the evidence base for long-term outcomes is limited, with most studies being project-specific and lacking rigorous longitudinal follow-up. There are currently no published or ongoing large-scale, longitudinal studies or national registries in Afghanistan that systematically evaluate the impact of telemedicine on health outcomes, cost-effectiveness, or overall health system performance [47,49]. To address these critical gaps, future research should prioritize rigorous longitudinal evaluations of telemedicine interventions, focusing on their effectiveness in managing chronic diseases while building on empirical evidence established in other contexts [50]. It is equally important to explore strategies for integrating telemedicine within the existing health system to enhance sustainability and ensure continuity of care. Comprehensive cost-effectiveness analyses should be undertaken to assess the financial viability and efficiency of telemedicine initiatives. Moreover, the development of national telemedicine registries would provide a structured framework for data collection, monitoring, and evaluation, facilitating informed policy and programmatic decisions. Implementation science research should also be emphasized to identify context-specific approaches that can optimize the adoption and scalability of telemedicine interventions. Ultimately, national coordination, coherent policy development, and sustainable financing mechanisms are essential to strengthen telemedicine infrastructure, promote equitable access, and improve health outcomes for all Afghans.

## Conclusions

Telemedicine in Afghanistan is an essential strategy for addressing the country’s healthcare challenges, particularly in reaching remote and underserved populations. Covering specialties such as critical care, mental health, ophthalmology, general medicine, and emergency services, it has improved access, quality of care, and cost-effectiveness. Persistent barriers, including limited technological infrastructure, gaps in provider training, regulatory shortcomings, and challenges integrating telemedicine with existing health systems, underscore the need for continued investment. By expanding specialty coverage, enhancing provider capacity, ensuring equitable access, and embedding robust monitoring and sustainable financing, telemedicine can significantly advance universal health coverage and improve health outcomes amid Afghanistan’s complex context of conflict, geographic isolation, and political instability.
